# Current Hospital Topics

**Published:** 1908-01-18

**Authors:** 


### HOSPITAL ADMINISTRATION.

### CONSTRUCTION AND ECONOMICS.

### CURRENT HOSPITAL TOPICS.

**The Cost per Bed and per In-patient.**

It has always been difficult, and previous to the general adoption of the Uniform System of Accounts it was practically impossible, to ascertain the average cost per bed and per in-patient at a number of separate hospitals for purposes of comparison. The difficulties were due to the fact that, in no two hospitals were the same items properly charged under the same headings in the accounts. This circumstance is fairly well illustrated by the first group of hospital statistics of an economic character which were prepared by the late Mr. Wilkinson, of St. Mary's, many years ago. The Uniform System of Accounts was originated at the Queen's Hospital, Birmingham, in 1868, and to-day the system is not only adopted by the King's Fund and the Hospital Sunday and Saturday Funds in London, but it is the plan on which the accounts are kept throughout the country in most well-administered hospitals. The third edition of the "Uniform System of Accounts for Hospitals, Charities, Missions, and Public Institutions" is now in the press, and will be shortly issued by The Scientific Press Ltd. We mention this fact as this is the season of the year when it is convenient to alter existing systems of accounts, and we much hope that all the larger hospitals in the United Kingdom which have not yet adopted the Uniform System may decide to do so at once. The system has now extended to the British Colonies, and is enforced in several of them by the Government. A uniform system of accounts is being generally accepted by the great hospitals in the United States which, in its essential features, follows the Uniform System adopted in this country, though it necessarily differs in detail owing to the American practice of making up their books in a somewhat special form. For practical purposes, then, it may be said that the whole of the principal hospitals throughout the English-speaking world will shortly keep their books upon practically the Uniform System, and that at last it will be possible for anyone who has a knowledge of hospital account-keeping to examine the economic working of each of these institutions, or of such as he may select, and to compare the cost of working one institution with another on a basis which will give weight and authority to the conclusions drawn from the figures.

The Uniform System when adopted cannot, of course, prevent the effect of special conditions as affecting the cost of maintenance to be met with in certain hospitals. Mr. Harry Johnson, the Secretary of the Leicester Infirmary, has written an interesting letter which well illustrates the necessity for great care in drawing any comparisons between the expenditure of one year and another. For instance, the actual figures giving the actual cost of each in-patient, and the average cost per day of a given year may be absolutely correct, but the higher or lower cost in a particular year may be due to temporary causes not likely to recur. Thus at Leicester last year rebuilding operations and extension works were in progress, and twenty-four beds were temporarily closed, reducing the number of beds available from 200 to 176. A reduction of one-eighth of the beds necessarily had the effect of increasing the average cost for this particular year, and so the figures for that year are misleading as they stand, for they can form no basis for any real comparison of the average cost. At Leicester, where the pressure on the beds is always great, arrangements had to be made to provide maintenance for the urgent cases, with the result that the average stay of each in-patient was diminished last year, and in this way the actual number of in-patients admitted to treatment was only reduced by fifty-four as compared with the previous year.

It is not uncommon, where the managers are inexperienced or careless, to issue an appeal informing the public that if a given sum is not provided the number of beds available for patients must be considerably reduced. In practice no such reduction in the number of beds can materially reduce the cost of maintenance of the whole establishment, seeing that practically the only possible reduction which results is in the case of provisioning.

Again, where the administration is careful and intelligent the upkeep both in stores and other requisites is well maintained, with the result that, as at Leicester, although there are only 200 beds available at the moment, when the new buildings are completed they will be increased to 300 beds, a number which the actual equipment of the hospital at the present time will be able to provide for without any additional outlay. This interesting result is due to the foresight of the managers in making adequate boiler and heating accommodation, with a sufficiently extensive kitchen and all that appertains to its efficiency, when they laid down the original plan. They have, further, been liberal in their arrangements as to the administrative staff, so that the additional cost of maintaining the extra hundred beds when they come into use will be materially reduced. Some extra addition to the cost of working may be attributed to the special system under which the out-patient department, with a separate dispensing staff and dispensary, and a new operation theatre, is organised. Then, too, the large nursing home of the infirmary is situated two miles away, and the cost of conveying the nurses to and from the infirmary amounts to £150 per annum, in addition to the upkeep of a separate domestic staff. All these special arrangements at Leicester tend to increase the cost of maintenance, though they no doubt offer advantages which, in the opinion of the managers, render this extra expenditure justifiable and necessary. The Infirmary has long held a foremost place as a training institution for nurses, under the able administration of Miss Rogers, who has given the best years of her life to make this work a success. Leicester Infirmary too, is remarkable from the fact that its influence throughout the country is so great that Leicestershire is the one county in England where the cottage hospital movement has never been able to gain a substantial foothold.

